# Tracing gestation and lactation in free ranging gray whales using the stable isotopic composition of epidermis layers

**DOI:** 10.1371/journal.pone.0240171

**Published:** 2020-10-29

**Authors:** Michelle Gelippi, Brian Popp, Marco F. W. Gauger, Javier Caraveo-Patiño

**Affiliations:** 1 Centro de Investigaciones Biológicas del Noroeste, La Paz, B.C.S., México; 2 Department of Earth Sciences, University of Hawaii at Manoa, Honolulu, HI, United States of America; Senckenberg Gesellschaft fur Naturforschung, GERMANY

## Abstract

The isotopic composition of baleen whales’ epidermis structural layers can give information about dietary change over time. This study investigated if epidermis layers integrated isotopic values that record physiological changes from gestation to lactation. Epidermis tissues (n = 43) were collected from free ranging lactating female gray whale and calves during the beginning of three breeding seasons. Modelling of δ^13^C and δ^15^N values show intra- and inter-individual differences based on epidermal layers, age class and year of sampling. The isotopic composition of mother-calf pairs is correlated, and the estimates of the maximum mother-to-calf isotopic difference was ~1.4‰ for δ^13^C and between 1 and 1.5‰ for δ^15^N values. Change in δ^15^N values among epidermal layers in calves was associated with the transition from fetus to consumption of maternal milk. It is here proposed when lactation influences calf epidermis, δ^15^N values decrease consistently from the outermost to the innermost layer. However, if a calf was born only few days before collection, epidermis integrates more variable δ^15^N patterns because gestation still affects the isotopic composition of the layers. The possibility of calculating mother-to-calf nitrogen isotope fractionation, and the regularity of changes between calf layer δ^15^N values, allowed results of an isotopic clock model to predict the age of each calf when sampled with its mother. This model has the potential to be a straightforward method to estimate the beginning of lactation, therefore calf birth date when direct observations are not feasible. The non-lethal remote collection of epidermis appears to be an effective tool for the study of the physiology of reproduction of baleen whales. The parallel study of the three epidermal structural layers highlighted the importance of considering the unique mother-calf pair physiological status at the time of sampling time when stable isotope results are interpreted.

## Introduction

Cetacean epidermis has three instead of the five layers normally found on terrestrial animals, which allows whales to better adapt to conditions found in marine environments including higher density, greater heat capacity and greater water pressure [1,2]. Cetacean epidermis [3] consists of an inner and outer layer named “stratum basale” (SB) and “stratum corneum” (SC), respectively, with the “stratum spinosum” (SS) in between, which is normally the most extensive of the three layers. In this continuously growing tissue, cells migrate from the SB, where they are formed, to the SC, where they undergo desquamation [2]. Furthermore, SB is anchored to the underlying dermal tissue by dermal papillae that project into the epidermis over the stratum spinosum [4].

Stable isotope analysis (SIA) of skin biopsies collected from free-ranging individuals has been widely used in the study of cetaceans [5]. Results of SIA can give insights into population ecology and dynamics of highly mobile marine predators [6]. Epidermis can act as an archival tissue that integrates the isotopic composition of animal’s diet over some period of time [7]. The time required for the isotopic composition of a tissue to reach 95% and 50% of the steady-state isotopic composition of the diet are known as “full isotopic turnover” and “isotopic half-life (t^1/2^)”, respectively, and are expected to be influenced by tissue growth, catabolic turnover and body mass [7,8]. Most studies have used epidermal tissue carbon and nitrogen isotopic composition to infer feeding ecology [9–14]. δ^13^C values can identify dietary carbon sources and potentially animal movements and δ^15^N values can indicate possible prey items and trophic linkages [15,16].

Recent results of SIA on epidermis strata subsamples of blue [13], and of humpback, sperm and fin whale [14], suggest temporal variations are recorded in discrete layers. In those studies, differences in δ^13^C and δ^15^N values across epidermal layers were interpreted to represent temporal change and were used to infer intra-specific shifts in foraging preferences and seasonal and regional movements. Particularly, the isotopic composition of SB reflects animals’ diet at the time of sampling, while that of the SC integrated the isotopic composition of past diet [13]. For all species investigated, inter-layer δ^15^N values differences appear to follow clearer patterns than δ^13^C values. This was justified by the greater potential of nitrogen compared to carbon to integrate differences between isotopic compositions of prey from different areas. The precise time period represented by each epidermal layer, however, it is not well known and could vary according to factors such as species and environmental conditions [14]. In addition, when intra-layer differences in δ^13^C and δ^15^N values were compared in stranded striped dolphins (*Stenella coeruleoalba*), bycaught common dolphins (*Delphinus delphis*) [17] and stranded gray whales (*Eschrichtius robustus*) [18], no significant differences were found, and authors assumed isotopic homogeneity within those species epidermis.

Based on this information, it is unclear if changes in δ^13^C and δ^15^N values across whales’ epidermal layers could be used to estimate how long ago an animal changed diet and if different layers record and integrate movement patterns or changes in metabolic and nutritional conditions. Nutritional and physiological status, such as gestation, lactation, and nutritional stress can affect the isotopic composition of mammals [5,6,19,20]. In marine mammals, the effects of pregnancy and lactation on stable isotopic compositions are poorly known, with most studies available for pinnipeds [21–28] and only few for baleen whale species [6,29,30]. Among this research, only one study focused on humpback whale (*Megaptera novaengliae*) epidermis (although structural layers were not taken into consideration) to evaluate the effects of pregnancy [6], and another on the epidermis of two hunted bowhead calves [30]. The two sampled bowhead calves were found with remains of fetal skin, appearing as patches along the body, while the main skin under the patches was new. Generally, isotopic fractionation is expected between mother-to-fetus/calf nutrients transfer, which is species-specific, tissue-specific and can be influenced by factors as breeding strategy and nutritional conditions [6,25,31]. Pregnant and lactating females can be “income breeders”, “capital breeders” or a mix between the two. Specifically, income breeders maintain a regular diet through pregnancy and lactation, capital breeders fast through the two physiological stages and the last group of animals are income breeders during pregnancy and capital breeders during lactation [32]. Pinnipeds are either “income breeders” (e.g. fur seals and sea lions [22,26,27,33]), or both “income” and “capital” breeders (e.g. elephant seals and grey seals [21,24,25,28]). On the other hand, most baleen whales are capital breeders and, in a theoretical framework of fasting, mother’s δ^13^C and δ^15^N values are expected to decline during pregnancy, because of lipid mobilization, increase of protein synthesis and decreases in nitrogen excretion [6,34]. Simultaneously, the fetus feeds on placental blood, rich in maternal proteins, which leads to an enrichment of ^15^N in the fetus relative to placental blood and to a balance between the tissues of the mother-fetus pair [29]. Little is known about mother-to-fetus isotope fractionation in capital breeding marine mammals. The only available results for a whale species, the fin whale, report that fetuses δ^13^C and δ^15^N values were about 1.13‰ and 1.5‰ higher than those of the respective mothers between muscle tissues [29]. In pinnipeds, red blood cells and serum of northern elephant seals did not differ in their δ^13^C values between mothers and fetuses, whereas the δ^15^N value of the mothers was on average 0.7‰ lower than the fetus [21]. In southern elephant seals whiskers [24], the isotopic composition of mothers and fetuses did not appear to be in steady state during gestation, however, both δ^13^C and δ^15^N values were reported to increase in fetuses and decrease in mothers with time during pregnancy. In grey seals, fetus red blood cells and serum δ^13^C values were lower than those of their mothers (of ~0.1‰ and 0.8‰, respectively), whereas fetus δ^15^N values were higher (of 2.4‰ and 1.3%, respectively) [25]. When lactation begins, isotope profiles are expected to change from those recorded in tissues during gestation [5]. Borrel et al. [29] reported significantly lower δ^13^C values in pregnant relative to lactating fin whales, and no significant differences were found in δ^15^N values. Habran et al. [21] found significantly higher δ^13^C values in serum of lactating female northern elephant seals sampled 5 days after parturition (assumed to reflect gestation) compared to 22 days after birth (assumed to represent lactation), but no significant differences were reported for δ^15^N values. On the other hand, in lactating grey seal serum, δ^13^C appeared to decrease and δ^15^N values to increase significantly between beginning and end of lactation (about 15 days apart) [25]. In baleen whale calves, decreases in both δ^13^C and δ^15^N values were found from fetal to calf epidermis in two bowhead whale specimens, although after weaning δ^15^N values of calf epidermis increased [25]. In the offspring of pinnipeds, northern elephant seal red blood cell δ^15^N values were reported to increase significantly between day 5 and 22 after birth, while serum δ^15^N values appeared to follow an opposite decreasing trend for the same time frame [21]. The δ^13^C values of whiskers in the offspring of southern elephant seals [24], declined during lactation relative to the end of gestation, and δ^15^N increased significantly. Conversely, stable isotope patterns of both red blood cells and serum did not change due to lactation in grey seals pups [25]. Mother-to-calf differences in δ^13^C and δ^15^N values during lactation has only been investigated in pinnipeds [21–23,25,28], because they typically breed on land and tissue samples can be collected directly for both mother and pup. These studies focused on elephant and grey seals that capital breed while lactating [21,25,28]. Whole blood, red blood cells and serum δ^13^C and δ^15^N values were reported to be generally higher in pups than in mothers at the final stage of lactation [21,25] and at weaning [28] of both species. On the other hand, grey seal pups serum δ^13^C values and hair δ^13^C and δ^15^N values were found to be significantly lower than those of their respective mothers’ tissues [25]. Consequently, the direction of change in mother and calf δ^13^C and δ^15^N values during pregnancy and lactation can differ depending on species and tissue analyzed.

The eastern gray whale is a good model animal to study because, like other baleen whales, it is a capital breeder and its migration patterns and feeding habits are well documented [30,35,36]. Gray whales are expected to feed in Arctic grounds during summer months and migrate to breeding lagoons in Baja California Sur, Mexico, during fall and winter. Pregnant females undergo a gestation period that lasts 11 to 13 months before giving birth in their calving grounds typically between late December and early March [35]. Thousands of individuals occupy the breeding lagoons, which include mainly mothers with newborn calves. They remain in those protected and calm waters until calves are ready to leave on the long journey to northern feeding grounds. These conditions provide the unique opportunity to collect tissue samples from free-ranging mothers and calves that would be otherwise challenging to sample during their migration along the North Pacific coast.

Here we investigated if the transition from gestation to lactation is recorded in the isotope composition of the three structural layers of gray whale lactating females and calves. Skin biopsies were collected from free-ranging and alive gray whale mother-calf pairs during the first two months spent in their main calving area, Ojo de Liebre lagoon. We determined epidermal inter-layer isotopic variability in mother and calf individuals, calculated the mother-to-fetus isotope fractionation directly from our sampled pairs and investigated if the expected dependency between mother and calf δ^15^N values [5] could be used to estimate calf birth-date using an isotopic clock model.

## Methods

### Ethic statement

The institution that granted the collection of skin biopsies was SEMARNAT (Secretaría de Medio Ambiente y Recursos Naturales) in México. Codes: SGPA/DGVS/0937, SGPA/DGVS/011543/17, SGPA/DGVS/010876/18 and SGPA/DGVS/12644/19.

### Epidermis biopsies

We analyzed the epidermis of 43 free-ranging female eastern gray whales (n = 19) and calves (n = 24) collected in Ojo de Liebre lagoon (Latitude: 27.75; Longitude: -114.25), Baja California Sur, Mexico, in February 2011 and 2018 and in January 2019. In 2011, 6 females and 5 calves were chosen randomly from gray whale mother-calf pairs that were forming typical temporary pods in the study area. Attempts made to obtain samples from both mother and calf of a single pair were however unsuccessful. Photo-ID and behavioral data were not collected. In 2018 and 2019 we applied focal sampling methodology [37] to mother-calf pairs that were not part of big pods and thus easier to identify, follow, and recognize for a long period of time (~3 hours per couple). We sampled 13 mother-calf pairs (2018: 5 couples; 2019: 8 couples) and 6 calves alone (2018: n = 4; 2019: n = 2) (attempts to collect tissue from their mothers were unsuccessful). The animals were photo-identified prior to each biopsy event to recognize animals and to avoid duplicate sampling.

During February 2011 and 2018 we sampled all organisms with a 5 m long stainless steel pole equipped with a modified stainless steel biopsy point [38]. We used a 20 cm long x 0.6 cm wide punch for the females, and a 10 cm long x 0.4 cm wide punch for the calves. In January 2019, mothers with a recently born calf were elusive and could not be easily sampled. For this reason, we collected tissues from lactating females with a crossbow armed with a stainless-still biopsy dart that was 2.5 cm long x 0.6 diameter. Calves were too small and moving unpredictably around the mothers to be sampled using a crossbow. Therefore, we focused sampling efforts on approaching the organisms as much as possible and the epidermis was collected with the long stainless steel pole used in 2011 and 2018.

All biopsies were taken when animals surfaced to breathe, in the emerged dorsal area between the dorsal and caudal fin. Once collected, tissue samples were wrapped in aluminum foil, placed inside sterile plastic bags, and stored on ice until we reached land (usually less than 5 hours). Thereafter, the biopsies were stored in liquid nitrogen and then frozen at -80°C. In between every biopsy event, punches were cleaned, sterilized, and stored in alcohol.

### Epidermis SIA

For each biopsy, we separated the epidermis from dermis and hypodermis at the Centro de Investigaciones Biológicas del Noroeste, La Paz, Mexico. The tissue was cut while frozen with a sterile scalpel.

Based on previous histological studies of other cetaceans’ epidermis [2,3,39], SS occupies almost all the central part of the tissue, and SC and SB compose its outermost and innermost sides, respectively. SC and SB were sampled by removing a subsample of the epidermis closest to the dermis and one at the outermost portion, respectively. The SS sample was obtained for material at approximately half of the total epidermis height. Total lipids were extracted from all samples (2:1 chloroform-methanol, 24 hours) prior to freeze-drying and isotopic analysis.

Stable isotope analyses were performed at the SOEST Stable Isotope Biogeochemical Facility of the University of Hawai‘i (UH) at Mānoa and at the U.C. Davis Stable Isotope Facility. Samples were powdered and weighed into tin cups (Mānoa: 0.5 mg ± 0.05; Davis: 0.1 mg ± 0.02), and their C and N isotopic composition was measured using a Costech elemental combustion system (Model 4010) coupled to a Thermo-Finnigan Delta plus XP isotope ratio mass spectrometer through a Conflo IV interface (Mānoa) and with an Elementar Vario Micro Cube elemental analyzer (Elementar Analysensysteme GmbH, Hanau, Germany) interfaced to an Isoprime VisION isotope ratio mass spectrometer (Davis). Glycine and tuna muscle tissue were used as reference materials to normalize the samples and to correct for instrument drift at Mānoa and Alfalfa flower, bovine liver, enriched alanine, glutamic acid and nylon 6 at Davis.

All isotope values are expressed in delta (δ) notation relative to V-PDB for carbon and atmospheric N_2_ for nitrogen. Accuracy and precision at UH were < 0.2 ‰, as determined from multiple laboratory reference materials extensively calibrated using National Institute of Science and Technology reference materials and analyzed every 10 samples.

### Statistical analysis

#### Evaluation of isotopic patterns between epidermis strata

We completed statistical analysis and graphical visualizations with the statistical software package R version 3.5.0 for Windows (R core Team 2017). Probability values < 0.05 were considered to be the critical statistical level of significance.

The investigation of marked subepidermal cells in two bottlenose dolphins [39] and in one beluga whale [40] showed that they move along the SB between 10 and 20 days after formation, in around 50 days they reach the tip of the SS and complete cells’ lifespan is concluded in 70–75 days in the SC. When new epidermal cells form, their isotopic composition reflects all the constituents present in the body blood stream at that specific time [41]. The most thorough controlled feeding experiment on cetaceans was on bottlenose dolphins [11] and determined the isotopic half-life of the SC to be 24 ± 8 days for carbon and 41 ± 16 days for nitrogen; complete turnover rate in the SC was 104 ± 35 days for carbon and 180 ± 71 days for nitrogen. Estimate of δ^15^N values for full incorporation rate of blue whale epidermis [13] was of 163 ± 91 days, which was comparable to that reported for the bottlenose dolphin epidermis, despite the great difference in body size between the two species. Assuming constant cell production and desquamation, each epidermis layer can undergo near complete nitrogen isotope turnover between 54±53 and 69±49 days [14]. Based on all this information, the isotopic values of each structural layer were assumed to represent different time periods. Calf epidermis δ^13^C and δ^15^N values depend on those of the mother, because both the fetus and the calf feed directly on mother’s tissues. Previous observations [35,36] suggest the maximum number of mother-calf pairs was expected to be reached in the breeding ground during the second half of February. However, at sampling, calf birth date was unknown. It is unknown whether measured isotopic composition represents comparable physiological conditions or if each individual integrated differently the effects of gestation and lactation.

We used linear mixed effect models (“*lmer*”) to test differences in δ^13^C and δ^15^N values in epidermal layers (lme4 package for R). The repeated measure design of individual’s epidermal layer isotopic values and the link between calf and mother isotopic values due to gestation and lactation were set as random effects. The values were allowed to vary within the boundaries of an individual ID and within the boundaries of mother-calf pairs IDs (1 | ID_mother-calf_/ID_layers_, [42]). Additionally, “year” (2011/2018/2019) was included as a further random effect. Fixed effects were “layers” (SC/SS/SB), “age” (mother/calf), and the interaction between “layers” and “age”. Model parameterization followed a forward model selection, where random effects were included before including fixed effects. The most parsimonious model was selected according to the lowest AICc value and AICc weights. AICc was chosen as the number of datapoints was relatively small (n = 127). Variables were tested for multicollinearity and residuals plots were visually inspected to detect possible deviation from homoscedasticity and normality (“performance” package for R). Then, models were visualized using simulated data that included confidence intervals estimated by bootstrapping (“bootMer” function of package lme4). Partial η^2^ and its confidence intervals were calculated to assess the effect size of the coefficients (“effectsize” package for R). Estimated marginal means (EMM, “emmeans” package for R) were computed and p-values adjusted for multiple comparison by Tukey. This post-hoc analysis was performed to estimate layer specific differences in mothers and calves.

Mother-calf pair epidermal layer δ^13^C and δ^15^N values were analyzed with paired t-tests. Mother and calf epidermis mean δ^13^C and δ^15^N values were compared, then the isotopic composition of each epidermis layer. Data normality was checked using the Shapiro-Wilk normality test. If data were not normally distributed, we used the paired two-samples Wilcoxon test, specifically the Wilcoxon Rank Sum and Signed Rank tests (“exactRankTests” package for R).

#### Assessment of gestation versus lactation in the different strata

Generally, after a change in the isotopic composition of a diet, the isotopic composition of a tissue changes with time. This change can be described by an exponential decay curve [43]:

$$\delta t=(\delta (0)-\delta (\infty ))e^{-\lambda t}+\delta (\infty )$$ (1)

where δ(0) is the isotopic value of a tissue at steady state with the old diet, and δ(∞)is the isotopic value of the tissue at steady state with the new diet. The parameter t represents time (in days) since the animal changed its diet, and λ is a data-derived first-order rate constant. If one knows the isotopic half-life (*t*^*1/2*^), defined as the time required for a certain isotope to reach the mid-point between steady state with the old and new diets [44], then λ can also be calculated from rearrangement of Eq 2 [45,46]:

$$t^{1/2}=ln(2)/\lambda$$ (2)

In this study, we assumed that the δ^15^N values of calf epidermis layers in steady state with nutrition supplied during gestation (*δ*(0), old diet) is different from the δ^15^N values of calf epidermis layers in steady state with nutrition from lactation (*δ*(∞), new diet). Therefore, the timing of this dietary change can be interpreted as an estimate of the time of calf birth. Depending upon the timing of calf birth, dietary changes in δ^15^N values may visible in different epidermal layers. If, for example, a calf was born just prior to sampling, one could expect that only the newest epidermal layer (SB) would integrate nutritional inputs from lactation. On the other hand, if a calf were several weeks old at the time of sampling, the change to nutrition from lactation would be recorded in the SS and SC, or SS only. Here, we propose the use of Eq 1 as a possible way to determine the timing of calf epidermis layer reflecting the nitrogen isotopic composition of maternal milk. We rearranged Eq 1 solving for duration since birth based on the epidermis δ^15^N values of each calf-mother pair:

$$t=\frac{ln\frac{\delta (0)-\delta (\infty )}{\delta (t)-\delta (\infty )}}{\lambda }$$ (3)

We calculated λ from Eq 2 using a nitrogen turnover rate or *t*^*1/2*^ of 42 ± 16 days [11].

Nitrogen isotope fractionation between mother and offspring is inconsistent between species [47], however, common patterns are recognized. We propose here two possible ways to calculate mother-to-fetus nitrogen isotope fractionation based on SIA of epidermis layers. Both are based on gray whale life history, where calf birth is known to occur between mid-December and mid-February [35]. The first method takes into account epidermal cell turnover rates [39,40] and full δ^15^N turnover [11,13], and SC δ^15^N values of both mother and calf are considered to represent always full gestation (*δ*(0)), independently from calf birth. On the other hand, the second method relies on the trend in δ^15^N values measured here among organisms’ epidermal layers and it assumes that full gestation effects could be visible in any layer as a function of the time since calf birth. A decline in δ^15^N values is indeed expected in mothers from conception to parturition, and the speed of these changes appears to increase together with mother and fetus weight gain [6,48]. If this is true, the mother-to-fetus nitrogen isotope fractionation is expected to reach its maximum shortly before birth. In the first case, mother-to-fetus isotope fractionation is calculated simply by subtracting mother SC δ^15^N values from calf SC δ^15^N values. In the second case, the highest δ^15^N value of mother’s epidermis is subtracted from the highest value of the corresponding calf, independent of the layers where they were found. Finally, for each method, we assume that the largest mother-to-fetus nitrogen isotope fractionation value measured approximates calf *δ*(0) as:

$${\delta (0)}_{calf}={\delta (0)}_{mother}+fractionation\;value\;(‰)$$ (4)

*δ*(∞)was calculated by the rearrangement of Eq 1:

$$\delta (\infty )=\frac{\left(\delta (t)-\delta (0)e^{-\lambda t}\right)}{1-e^{-\lambda t}}$$ (5)

δ(*t*) could not be represented by any of the δ^15^N values recorded in the sampled calf epidermis, because we did not know *t*, or calf age, the main unknown variable of our model. Jenkins et al. [47] present evidence of a correlation between mother and calf δ^15^N values recorded at a specific time after birth and consistent between different species. In 11 terrestrial mammals of different size and diet specializations approximately 15 days after birth, mother’s blood components (total red blood cells and plasma) has been shown to continue to reflect *δ*(0) values, and calf blood incorporates δ^15^N values that are about equal to those of its mother *δ*(0) [47]. In this study, we did not analyze blood. However, total red blood cells and plasma and epidermis are known to have similar δ^15^N turnover rates [11,41,47] and we assume these apply to a marine mammal. Based on observations of terrestrial mammals, we assume that convergence of mother-calf δ^15^N epidermis values is an indication of the 15 days after calf birth. In other words, when mother-calf δ^15^N values are equal it indicates calf *δ*(*t*) of 15 days and we can apply Eqs 2 and 4.

For each calf, the calculated *δ*(0) and *δ*(∞)values were used to create the exponential decay curve representing the change from gestation to lactation inputs. In addition, the age of each epidermis layer was calculated independently using Eq 3, and the expected δ^15^N values of calf epidermis at *t*^*1/2*^ were calculated using Eq 1. Finally, calf birth date was estimated by subtracting the age of SB from the date when calf biopsy was collected.

## Results

### SIA of epidermis strata

**Fig 1:**
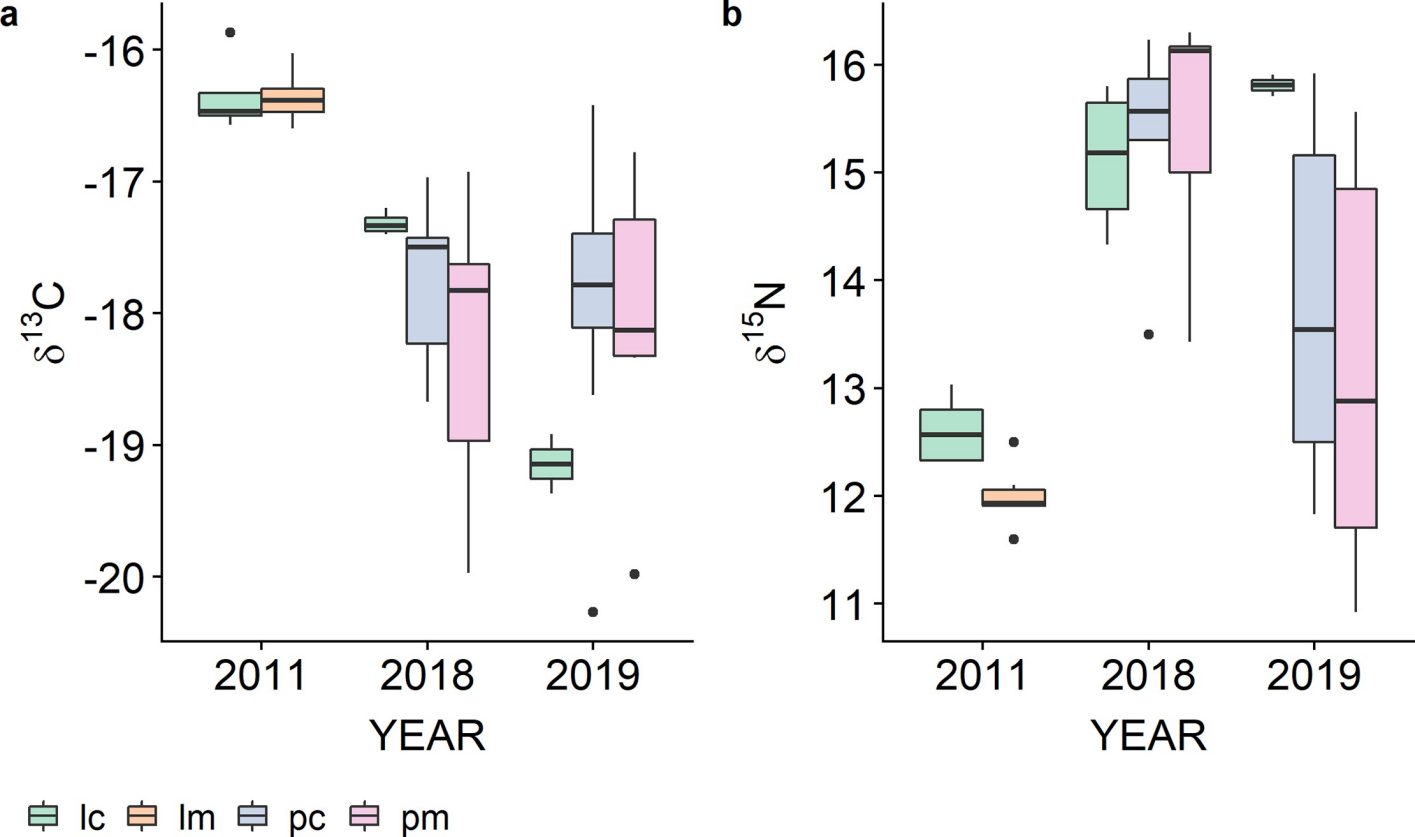
Boxplots of the mean δ^13^C and δ^15^N values of gray whale mothers and calves epidermis collected in February 2011, February 2018 and January 2019. Results are divided between lactating females and calves sampled alone, indicated as “lm” (lonely mothers) and “lc” (lonely calves), and mothers and calves individuals sampled with their pairs, indicated as “pm” (pair mothers) and “pc” (pair calves).

**Fig 2:**
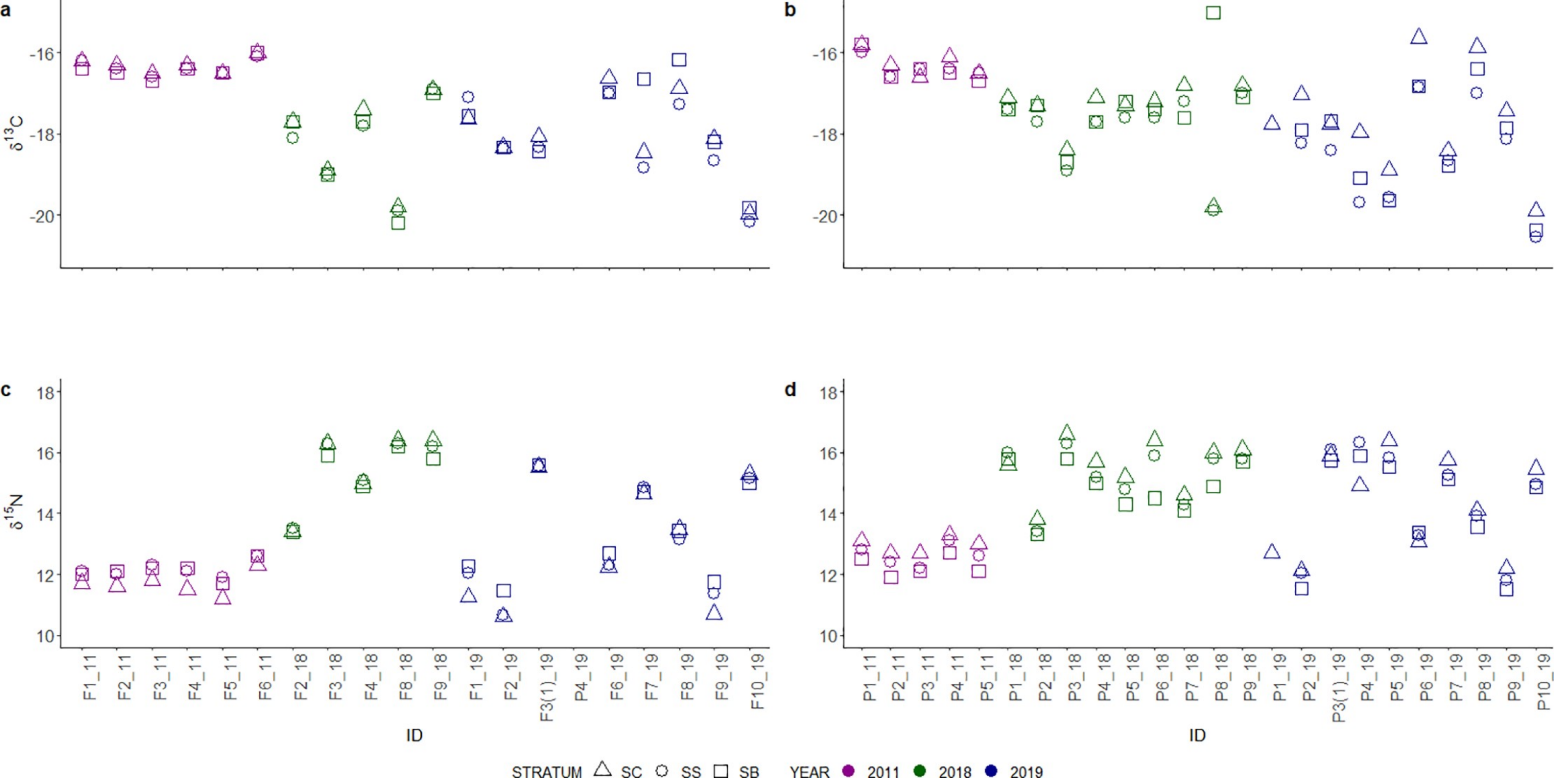
Intra-individual differences among epidermis layers normalized to SC for δ^13^C and δ^15^N values. Standard errors were represented by blue-shadowed lines.

Epidermis δ^13^C and δ^15^N values of all gray whale together ranged from -15‰ to -20.5‰ and from 10.6‰ to 16.6‰, respectively (S1 File) and mean epidermis values ranged from -15.9‰ to -20.3‰ for δ^13^C and from 10.9‰ to 16.3‰ for δ^15^N (Fig 1). The magnitude of the differences in the isotopic composition of the structural layers are shown in Fig 2 for each mother and calf specimen. The trend of change in δ^13^C values (Fig 2A) is variable in 2011 and 2018 mothers and in 2011 calves, while in all 2019 mothers, but one, and in 2018 and 2019 calves δ^13^C values decreased from SC to SS, to then increase again in the SB. Differences among δ^15^N values (Fig 2B) are variable for mothers, conversely calves mostly show decreases in δ^15^N values from SC to SB. However, the δ^15^N values of a few specimens increased from SC to SS and then decrease in the SB layer. One calf collected in 2019 had δ^15^N values that increased from SC to SB.

**Fig 3:**
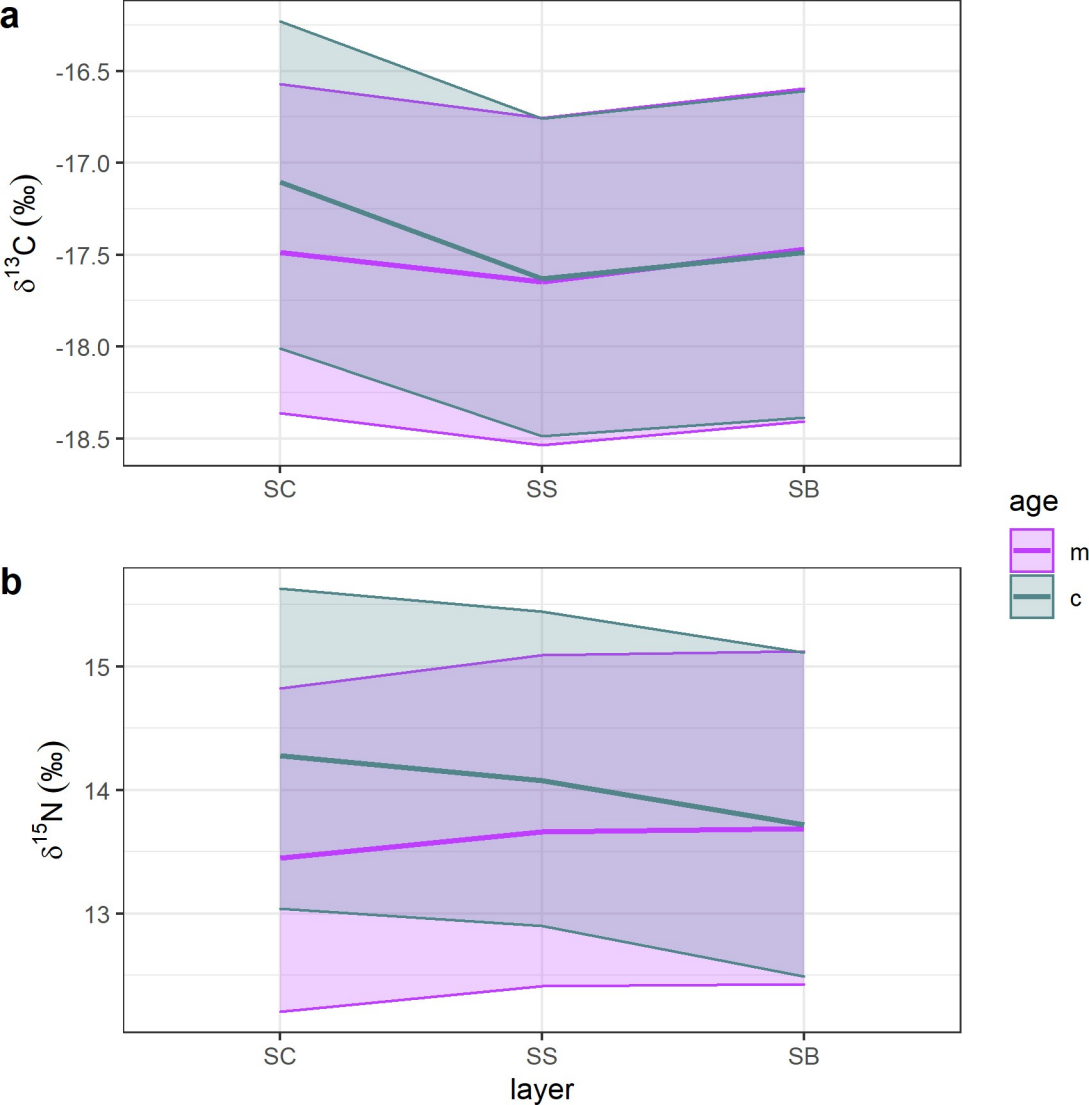
Top selected linear mixed effect model for δ^13^C (a) and δ^15^N (b) patterns. For both models, fixed effects were “layer”, “age” and the interaction “layer*age”. Random effects were “individual ID”, “mother-calf pairs IDs” and “year” (2011/2018/2019).

**Fig 4:**
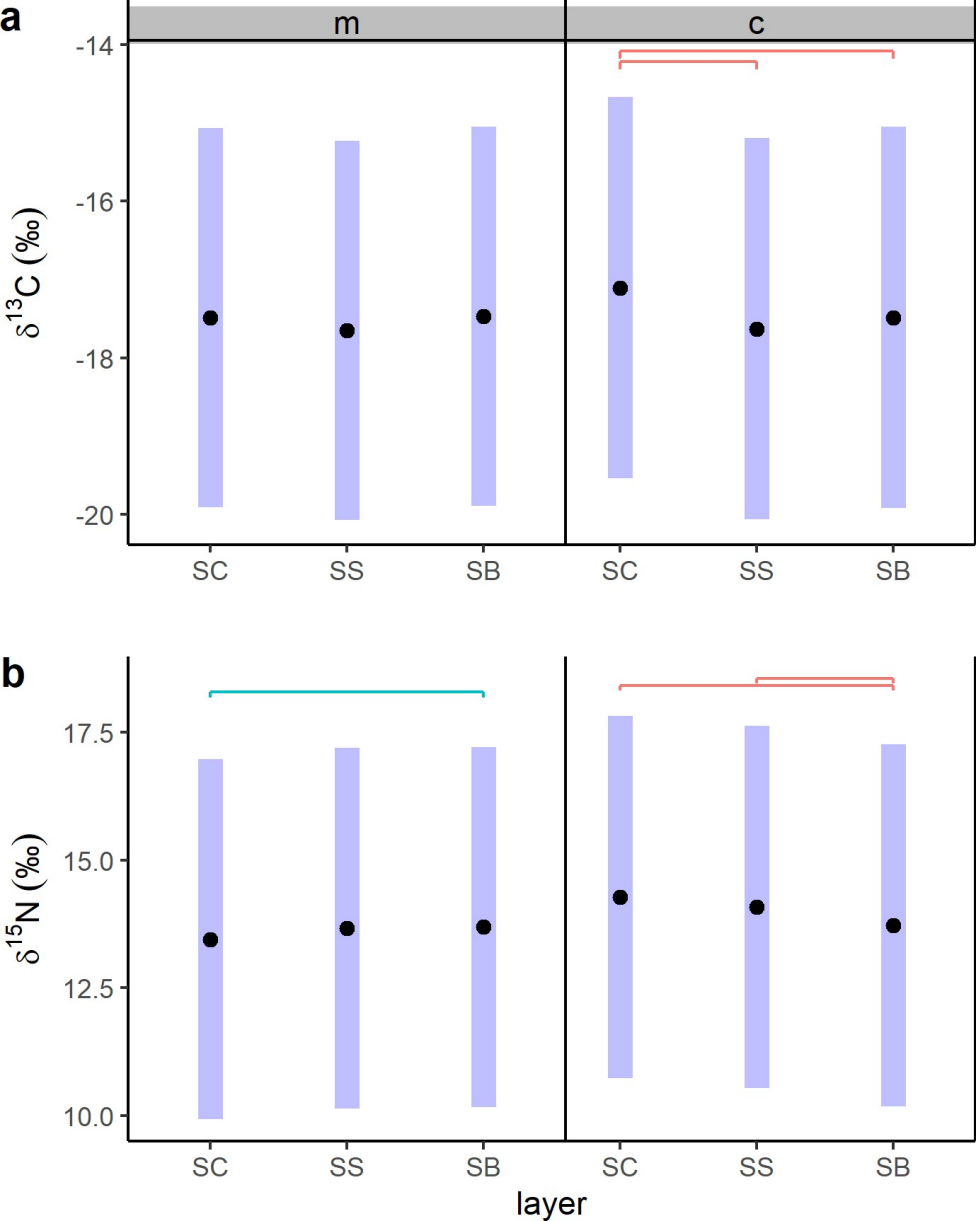
Post-hoc analysis results for linear mixed effect model predictions of δ^13^C (a) and δ^15^N (b) values based on “layer” (SC, SS and SB) within each “age” (m = mothers; c = calves). Significant differences between groups are indicated by upper lines.

The top-selected linear mixed effect models showed that changes in δ^13^C and δ^15^N values were most influenced by “layer”, “age” and by the interaction “layer*age” (Table 1; Fig 3; S1 File). One outlier (calf P8_18) was detected and removed from the best fitting δ^13^C model. No apparent problems were detected due to multicollinearity. Visual inspection of residuals did not reveal any obvious deviation from homoscedasticity or normality. The 95% confidence intervals predicted for δ^13^C patterns are high for both mothers and calves (Fig 3A). Mother δ^13^C values appeared to vary little among epidermal layers, and calf δ^13^C values were higher in the SC than in the other two layers. Post-hoc evaluation (Fig 4A) confirmed that δ^13^C values did not vary significantly among mothers’ epidermal layers, and that calf SC δ^13^C values differed significantly from those of SS and SB. Mother and calf patterns appeared to differ most in their SC, and then to converge in their SS. Similarly to δ^13^C predictions, estimated 95% confidence intervals for δ^15^N patterns were high (Fig 3B). Mother and calf δ^15^N values increased and decreased, respectively, from SC to SB. Post-hoc analysis (Fig 4B) indicated that mother δ^15^N values differed significantly between SC and SB, and that calf SB δ^15^N values differed significantly from both SC and SS. The largest difference in δ^15^N values was found between mothers and calves SC, and their δ^15^N values tended then to converge in the SB.

**Table 1:** Top-selected linear mixed effect model results for both δ^13^C and δ^15^N values.

	*Estimate*	*CI low*	*CI high*	*t value*	*df*	*Partial η*^*2*^	*CI low*	*CI high*
***δ***^***13***^***C***								
Intercept	-17.11	-18.37	-15.8	-37,26	2.94	0.924	0.904	0.938
Layer (SS)	-0.52	-0.7	-0.35	-5,979	82.28	0.239	0.134	0.344
Layer (SB)	-0.38	-0.55	-0.21	-4,363	82.28	0.143	0.058	0.244
Age (m)	-0.38	-0.6	-0.16	-3,467	46.17	0.095	0.027	0.189
Layer (SS) * Age (m)	0.36	0.11	0.62	2,813	81.59	0.065	0.011	0.150
Layer (SB) * Age (m)	0.4	0.14	0.66	3,104	81.59	0.078	0.017	0.167
***δ***^***15***^***N***								
Intercept	14.28	12.41	16.1	21.66	2.94	0.8	0.75	0.84
Layer (SS)	-0.2	-0.37	-0.04	-2.41	85.2	0.05	0.004	0.12
Layer (SB)	-0.56	-0.73	-0.39	-6.72	85.2	0.28	0.17	0.38
Age (m)	-0.83	-1.09	-0.57	-6.36	35.71	0.28	0.15	0.36
Layer (SS)* Age (m)	0.42	0.17	0.66	3.35	84.74	0.09	0.02	0.18
Layer (SB) * Age (m)	0.8	0.55	1.05	6.45	84.74	0.26	0.16	0.37

**Fig 5:**
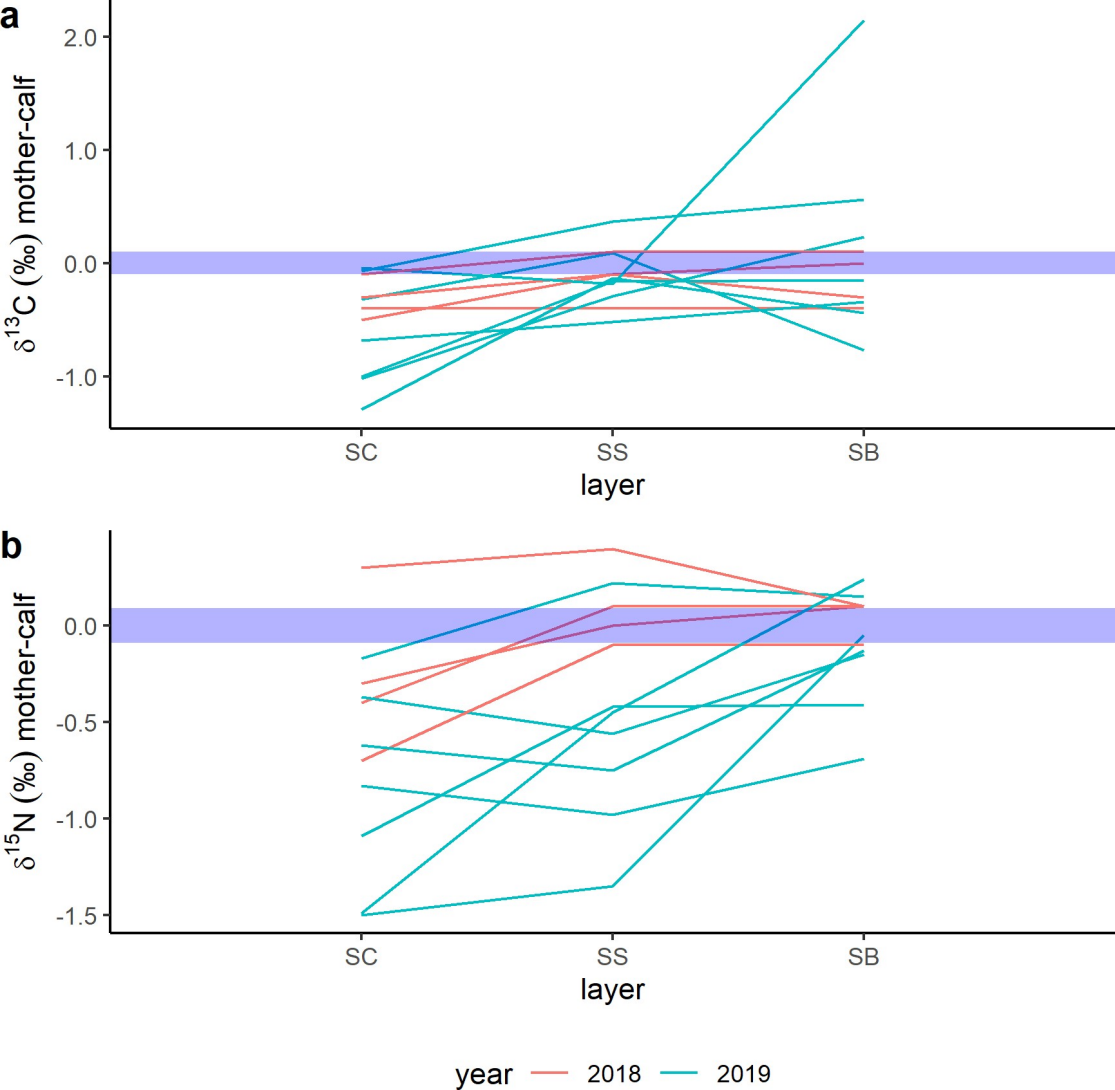
Mother-calf pairs differences among epidermis layers normalized to the mean of all three layers for δ^13^C and δ^15^N values. Standard errors were represented by blue-shadowed lines.

Finally, δ^13^C and δ^15^N values were analyzed for mother-calf pairs only. Shapiro-Wilk normality test indicated that mother-calf pair mean epidermis δ^13^C and δ^15^N values were normally distributed, while data normality was not confirmed for all isotopic values when the interaction “epidermal layers*year” was considered (S1 File). Paired t-test and paired two-samples Wilcoxon tests showed that the mean δ^13^C and δ^15^N values did not differ between mother-calf pairs, and that the mother-calf isotopic relationship was not influenced by “year” of sampling (S1 File; Fig 5).

### The isotopic clock model

**Fig 6:**
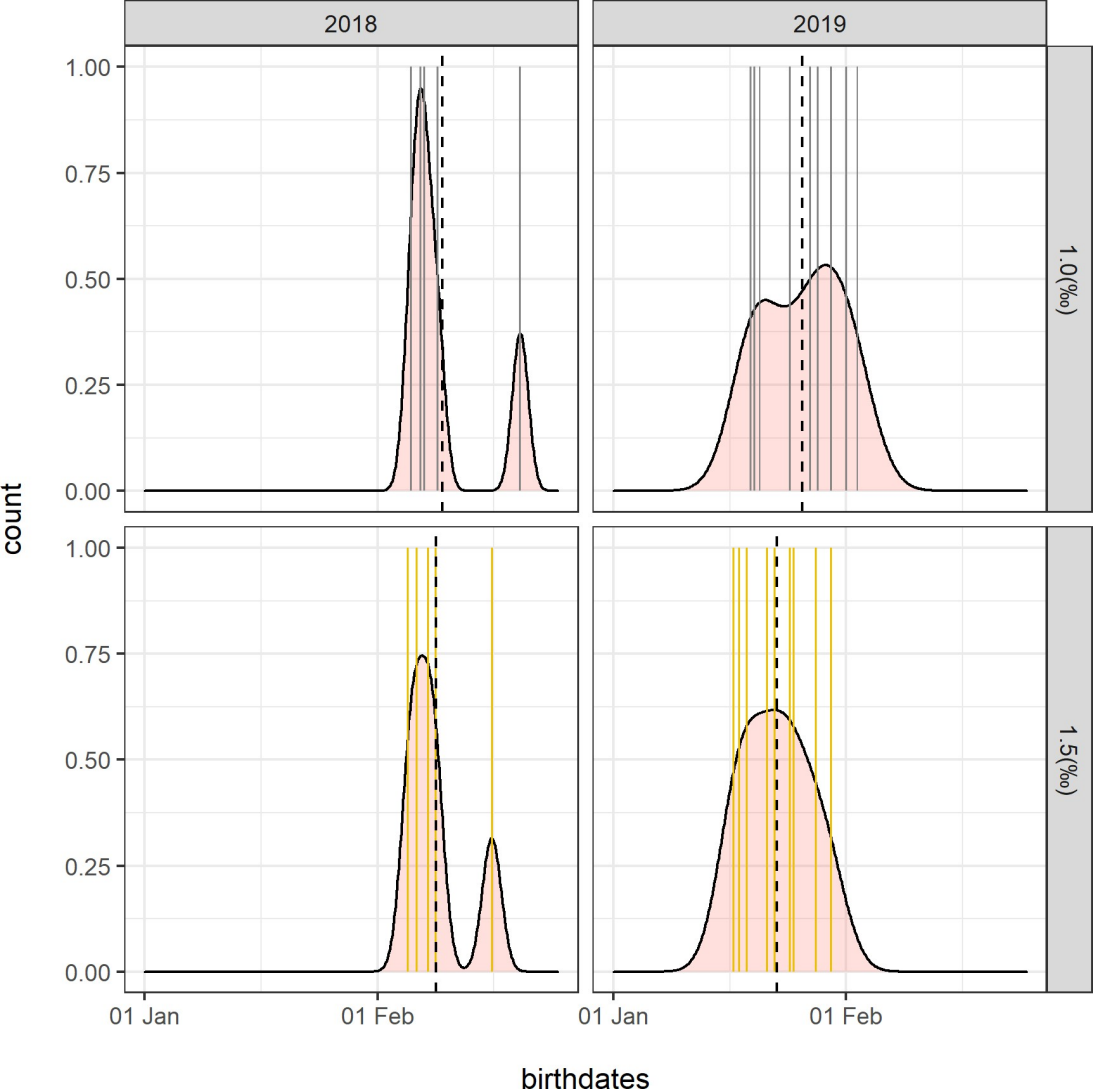
Density plot with counts, representing birth dates estimated for calves sampled with their mothers during January 2019 and February 2018. Different outcomes from the use of a mother-to-fetus nitrogen isotope fractionation index of ~1 ‰ (gray color) and 1.5 ‰ (yellow color) are shown. Black dotted lines indicate mean values.

A comparison of mother-calf pair δ^13^C and δ^15^N values between epidermal layers showed that nitrogen isotope composition varied more than carbon (Fig 5). The greatest difference between mother and calf carbon and nitrogen isotopic composition was expected when a calf was born close to sampling. Mother-to-fetus carbon isotope difference was calculated by subtracting the maximum δ^13^C_calf_ value from the minimum δ^13^C_mother_ value. Results indicated a maximum difference of 1.4‰ and a minimum of 0.2‰ (S1 File, Table). Mother-to-fetus nitrogen isotope difference was calculated for each couple using two different methods (S1 File, Table). “Method 1” results used SC (Δ)δ^15^N_Calf—Mother_, and “method 2” used the maximum (Δ)δ^15^N_Calf—Mother_ value. For mother-calf pairs where δ^15^N values decreased from SC to SB (all 2018 mother-calf pairs and 2 couples from 2019) the two methods gave similar results, because SC always recorded the highest values. For the remaining mother-calf pairs, “method 1” generally gave higher mother-to-fetus nitrogen isotope differences than “method 2”. The highest calculated isotope difference was 1.5 ‰ (“method 1”) and 0.9 ‰ (“method 2”). Given that both of these values were from mother-calf pairs sampled in January 2019 (S1 File, Table), which was the beginning of breeding time [35], we assumed they represented a good approximation of the gray whale mother-to-fetus nitrogen isotope fractionation during gestation. Mother-to-fetus isotopic fractionation was required to predict calf epidermis δ^15^N values when at steady state during gestation (*δ*(0)) (Eq 4), which was an unknown variable needed to predict calf age (Eq 3). To explore possible consequences of the use of different isotope fractionation values, we performed a sensitivity analysis of calf age calculations separately using values of 1.5 ‰ (“method 1”) and ~1 ‰ (“method 2”). Results of calf birth dates measurements are shown in Fig 6. Birth dates ranged from the 11^th^ of January to the 12^th^ of February using “method 1” and from the 19^th^ of January to the 20^th^ of February using “method 2”. Results were not significantly different for each method. Bimodal distributions of birth dates were found in 2018 using both methods and to a lesser extent in 2019 using “method 1”. The bimodality in birth dates found in 2018 using both methods resulted from a single calf with a calculated birth date in mid-February. During 2019, the distribution of birth dates covered a wider range of time regardless of the method used. Mean calculated birth dates in 2018 differed by only 1–2 days depending on the calculation method. Whereas mean birth dates in 2019 differed by 6 to 8 days depending on the calculation method used. Younger mean birth dates were found for “method 2” using a value of ~1‰.

**Fig 7:**
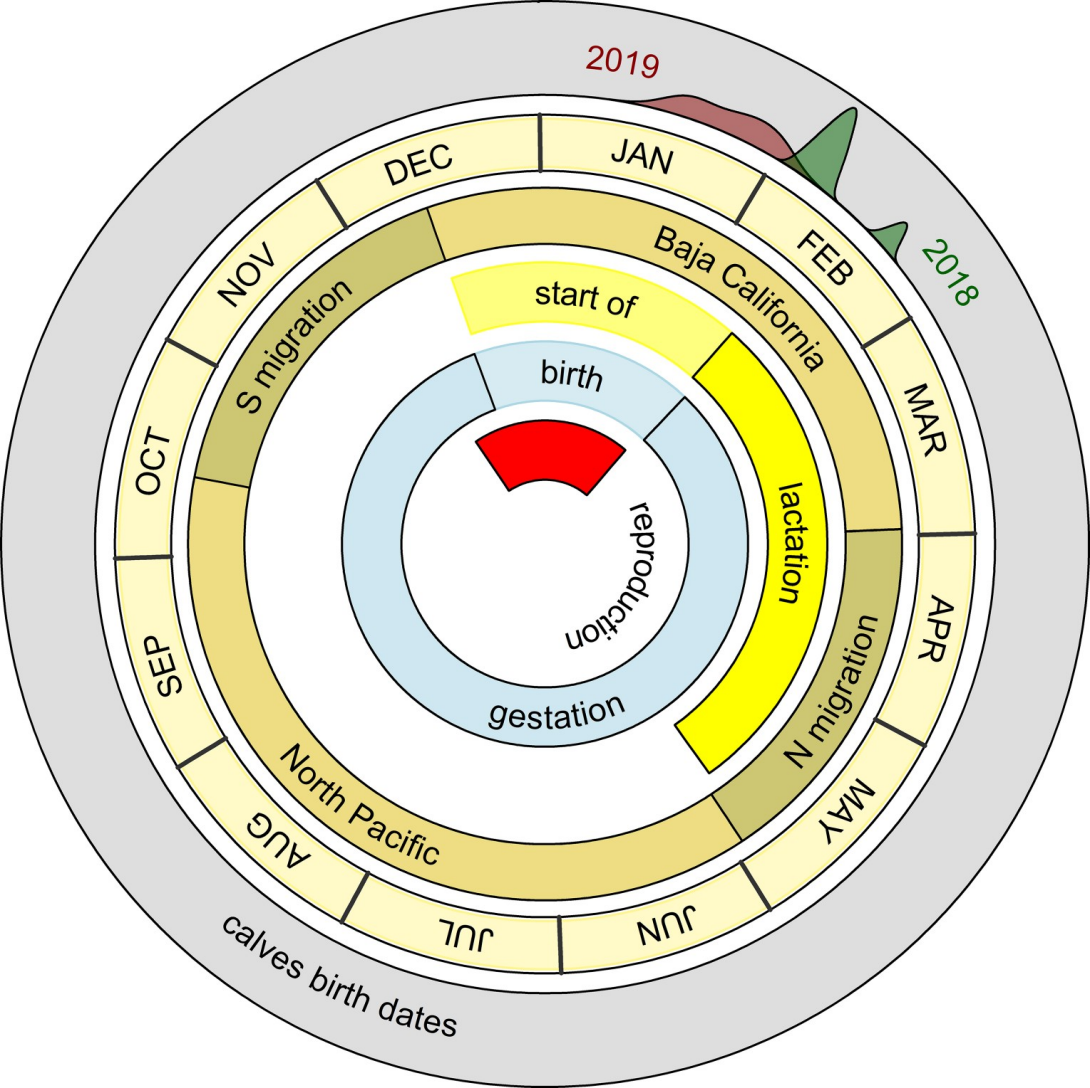
Circular plot representing calves’ birth date distribution in relation to the reproduction, gestation and lactation periods expected for the species [35]. Calves birth dates were calculated from the isotopic clock model (Eq 2), using the mother-fetus nitrogen isotope fractionation index of ~1 ‰.

Calves birth dates calculated using both the 1.5 ‰ and the ~1 ‰ mother-to-fetus isotopic differences agreed with known times for gray whale parturition. A timeline of gray whale reproduction, gestation and lactation periods expected for this species is shown in Fig 7. The range of birth dates was calculated using “method 2” (i.e, ~1‰). The predicted calf birth dates for 2018 and 2019 were compared with the times known for gray whale migration, for the residence in the breeding and feeding areas, and for lactation, gestation and reproduction [35]. At the time of sampling, calves from 2018 were 8 to 21 days (±16 days) old and calves sampled in 2019 ranged from 0 to 7 days (±16 days) old. Consequently, birth dates ranged from 5 to 16 February 2018 and 16–29 January 2019.

## Discussion

The potential of whale epidermal tissue isotopic composition to integrate physiological and nutritional effects was supported by the isotopic patterns found among gray whale lactating females and calves. The modelling of δ^13^C and δ^15^N values highlighted intra- and inter-individual differences, due to epidermis layers, age class, and year of sampling. The isotopic composition of mother-calf gray whale pairs did not differ, thus suggesting direct nutrient transfer from maternal to offspring tissues. Changes in δ^15^N values among mother-calf epidermal layers corresponded closely to the transition from gestation to lactation, which is a dietary change from placental blood to maternal milk in calves. Previous works [13,14] showed that differences in δ^15^N values among epidermal layers reflects dietary information, and this study is the first to use this information to investigate a baleen whale species during breeding. Predicted gray whale mother-to-fetus epidermis nitrogen isotope fractionation was similar to that reported in the literature for different tissues and different mammal species [21,29,48]. With this information and with available data on cetaceans’ epidermal isotopic turnover rates [11,13], it was possible to estimate calf epidermal layers age and to estimate the timing of the transition from gestation to lactation and, thus calf birth-dates.

### δ^13^C and δ^15^N patterns in lactating females and calves

By focusing on the collection of mother and calf individuals, we were able to determine a priori important characteristic that are difficult to detect during sampling campaigns of free-ranging animals, such as sex, age, and reproductive status. Data modelling highlighted the relevance of variables as epidermis layers (SC, SS or SB), age class (mother or calf) and the interaction between the two for the interpretation of SIA results for both carbon and nitrogen. Mean δ^13^C and δ^15^N values differed between organisms sampled during different years. On the other hand, δ^13^C and δ^15^N values changed among epidermal layers and age classes in similar ways and, consequently, year of sampling was not included in the best-fitted models as a fixed effect.

Prediction of mother-to-calf nutrient transfer from δ^13^C values is generally complicated by the high lipid content of maternal milk [5] and studies in this regard are not definitive. Newsome et al. [6] suggested a constant decrease in capital breeder epidermis δ^13^C values during pregnancy, and results based on a study of fin whale muscle tissues [29] indicated an increase in δ^13^C values during lactation. These changes were likely associated with lipid mobilization during gestation and lactation. During pregnancy, lipids are metabolized and used directly to provide energy during final gestation. On the contrary, during lactation, the production of lipid-rich maternal milk with low δ^13^C values would result in an enrichment of ^13^C in tissues proteins of females relative to gestation. Here, epidermis δ^13^C values both increased and decreased among layers of lactating gray whales (Fig 2A). Interestingly, δ^13^C values increased mainly in January (2019), when animals were supposed to have just started to lactate, while stronger inter-individual variability was found in February mothers (2011 and 2018). The only evidence reported in the literature for calves is from two bowhead whale specimens [30] and suggested a decrease in δ^13^C between fetus and calf, due to maternal milk consumption. In gray whale calves, only 9 out of 24 individuals had lower δ^13^C values in their SB (newest layers) compared to SC (oldest layer) (Fig 2A). The absence of clear trends in δ^13^C values among the epidermis of mothers and calves made it difficult to interpret our results. Inter-individual variability was confirmed by the high estimation of 95% confidence intervals of the top-selected linear mixed effect model for δ^13^C values (Fig 3A). Model and post-hoc predictions suggested that δ^13^C values changed significantly only between calf SC and SB, while in lactating females δ^13^C values changes were not significant. It is possible that mothers and calves were expressing differently the change from gestation to lactation, but further investigations are needed to clarify this hypothesis.

Results of linear mixed effect models of δ^15^N values allowed a more refined interpretation of data. In lactating females, mean and inter-layer δ^15^N patterns appeared to vary among years of sampling (Fig 1B and Fig 2B). The top-selected linear mixed effect model for δ^15^N possibly reflected this variability in the high estimates of 95% confidence intervals (Fig 3B). Year of sampling, however, was considered as a random effect only and model outcomes indicated that variability in δ^15^N values was mainly associated to inter-layer differences. This result, together with post-hoc estimates showing a significant change in δ^15^N values between SC and SB (Fig 4B), could indicate the effect of the transition from gestation to lactation on the nitrogen isotope composition of the epidermis of lactating females. It is known that year-to-year difference in environmental conditions can greatly influence the physiology of baleen whales, due to the fluctuation in prey quantity and quality prior to and during pregnancy [6]. Nitrogen isotopic composition of animal tissues can reflect nutritional stress, however, the extent of their usefulness is unclear. A previous study of nitrogen balance in muscle and baleen plates of fin whales indicated that only a severe condition of starvation would affect δ^15^N values in muscular tissues [19]. Simultaneously, the comparison between δ^15^N values recorded in the epidermis and muscles of gray whale specimens with good and poor body conditions [30] showed no increase in δ^15^N values in the skin of stranded animals, while it did in muscle tissues. Horstmann-Denn et al [30] speculated that structural proteins in the epidermis do not undergo catabolism during nutritional stress, whereas catabolism under these conditions produces an increase in the δ^15^N values of muscle. Based on these observations, we suggest that the inter-year and the inter-layer differences found in lactating gray whale epidermis δ^15^N values do not reflect nutritional stress. The indication of a year-to-year variation in epidermis mean δ^15^N values could reflect the fact that gray whale are a flexible feeder; thus, lactating females could have fed on prey from different trophic levels during gestation. In this work we did not estimate diet, however, it is possible that we sampled by coincidence animals coming from different feeding grounds, as for example the Bering Sea and the waters around Vancouver Island, British Columbia, where available prey species differ [49–52]. Furthermore, δ^15^N variability in lactating females could have been associated with the effect of different temperatures on epidermis. Temperature is indeed known to influence tissue catabolism rates, as proven histologically for the beluga whale [40], and to possibly slow nitrogen isotope incorporation, as for blue whales migrating in colder waters [13]. Those studies, however, analyzed animals that moved between habitats that differ greatly in their temperature ranges. Blue whale specimens, for example, were sampled along the North Pacific coast and in the Gulf of California, two basins systems with vastly different surface water temperature ranges. All our gray whales were instead moving along the North Pacific coast only, and water temperatures of summer feeding grounds and winter breeding lagoons differ by less than 5°C (NOAA). For all these reasons, we do not expect temperature to have influenced the nitrogen isotope composition of gray whale mothers.

Calf δ^15^N values differed significantly between layers, following a consistent decrease from SC to SB in 2011 and in 2018, although trends were more variables during 2019 (Fig 2B). Top-selected linear mixed effect model and post-hoc estimates confirmed a common decreasing trend in δ^15^N values from SC to SB for all the sampled calves (Fig 3B and Fig 4B). To the best of our knowledge, this is the second time that similar trends are reported for baleen whale calf epidermis. The first, involved the SIA of one hunted newborn bowhead calf [30], which epidermal fetal patches were enriched in ^15^N (δ^15^N = 14.8 ‰) relative to layers representing early life outside the maternal womb (δ^15^N = 12.4 ‰). Conversely, in other capital breeding marine and terrestrial mammal offspring, SIA do not unambiguously support the decreasing trend in δ^15^N values found here. In northern elephant seals, for example, a decrease in δ^15^N values was observed in serum but not in red blood cells between the initial and the final stage of lactation [21]. Conversely, in grey seals, red blood cells, serum and hair appear to get enriched in ^15^N during lactation [25]. In polar bears [31], SIA results appear to be individual-specific, where δ^15^N values can both decrease or increase in cubs plasma during lactation. On the other hand, grizzly bear offspring δ^15^N values are reported to decrease in their plasma during 3 months of lactation [47]. All these results highlight that no generalization can be made when different tissues, of different species, which undergo different fasting and lactation periods, are compared. Here, we propose the trend of change of δ^15^N values found in the epidermis layers of most gray whale calves could be associated with lactation effects. A similar interpretation was given by Horstmann-Dehn et al. [30] for the lower δ^15^N values found in the new epidermis of the bowhead calf compared to those of its epidermal fetal patches. On the other hand, when more variable patterns are recorded, as for some 2019 individuals, we suggest that calf birth occurred so close to sampling time that δ^15^N values most likely did not yet reflect lactation effects; an assumption that needs confirmation. Assuming gray whale epidermal cell turnover is similar to that of bottlenose dolphin [39] and beluga whale [40], maternal milk inputs should start to be recorded in calf SB δ^15^N values between 10 and 20 days after birth. Pregnant gray whales are expected to give birth in the breeding areas of Mexico from the end of December to the beginning of March [35], however, in 2019 gray whales were reported to arrive later than normal in the study area, and first newborn calves were only spotted starting in mid-January (unpublished data, Biosfera Reserve “El Vizcaino”). Based on these observations, it is plausible that calves collected in the middle of February 2011 and 2018 were older than those sampled in January 2019, and their epidermis δ^15^N values represent different periods of time, with respect to birth and the onset of nursing.

### Mother-fetus isotopic difference and estimate of calf birth date

The isotopic composition of fetal and newborn capital breeders is expected to be dependent on that of the mother [21,29,47]. Top-selected linear mixed effect models indicated that δ^13^C and δ^15^N values differed between mothers and calves always in their SC, and then isotopic values tended to converge in their SS and in their SB, respectively (Fig 3). Paired t-tests did not show significant differences in the mother-calf isotopic composition of combined epidermal layers as a function of year of sampling (S1 File). However, individual intra-layers differences normalized to SC (Fig 2) suggested 2019 mother-calf pairs had more variable isotopic patterns than those sampled in 2018. The discrepancy could be related to the different influence of gestation and lactation on mother-calf pairs sampled during January 2019 and February 2018. As previously discussed, it is possible that some 2019 calves were younger than those from 2018 and that the isotopic composition of their epidermis layers varied accordingly.

δ^13^C values are expected to decrease in mothers and increase in fetuses during pregnancy, and to increase in mothers and decrease in calves during lactation [6,29,30]. Accordingly, we used the minimum δ^13^C_mother_ and the maximum δ^13^C_calf_ values to estimate mother-to-fetus carbon isotope fractionation in the epidermis. The maximum values of 1.3‰, 1.4‰ and 1.4‰, were found in 3 mother-calf pairs from January 2019 and were higher than those found in fin whale muscle tissues, where the isotopic fractionation was of 1.1‰ [24]. We consider the differences between these results more related to sampling than to isotopic discrimination between tissues. We collected live and free-ranging gray whales, while Borrell et al [29] analyzed dead animals, for which the stage of pregnancy was unknown. δ^13^C values are expected to continue to decrease in mothers and increase in fetuses until the end of gestation [6], thus it is possible that fin whales did not reach that final stage when captured. Our results underline the importance of considering unique mother-calf pair physiological status at sampling time when results of SIA are interpreted. Future studies should focus on whether carbon isotope composition of mother and calf epidermis layers could be used effectively to detect the temporal change between gestation and lactation.

Calf-specific changes in δ^15^N values among epidermis layers was particularly useful to estimate mother-to-fetus nitrogen isotope fractionation and consequently, calculate calf birthdate. The direct observation of calf birth events is extremely rare for any free-ranging cetacean species. Here, we propose the use of a first order, one-compartment isotopic clock model [43,45,46] as a promising method to estimate time of birth. The isotopic clock model is normally used to estimate the time of a change in diet in animal tissues, under the assumption that the stable isotope ratio of the new diet input is constant [43]. Because we could not measure experimentally some of the parameters required by the model, we had to assume them based on the available literature. Our assumption are: 1) maternal milk has δ^15^N values that do not change with time; 2) δ^15^N values of calf epidermis at birth, representative of life in the womb, can be calculated from the observed mother-to-calf nitrogen isotope fractionation; and 3) 15 days after calf birth, δ^15^N values of mother and calf epidermis are equal.

The collection of maternal milk from baleen whale is logistically challenging and no literature data is available to determine if milk isotopic composition varies during lactation stages [53]. Available milk samples have been collected only from carcasses of females where calf day of birth and health conditions were unknown. Few studies calculated the isotopic profile of milk proteins in marine mammals [22,29,31] and results are not consistent. The only investigation of a baleen whale, the fin whale [29], analyzed milk samples obtained from carcasses of lactating females collected in the feeding, and not in the breeding grounds. Consequently, authors hypothesized that both δ^13^C and δ^15^N values could have already been influenced by prey inputs, thus no longer reflecting full fasting. During the first months of lactation, capital breeders produce milk that is composed of fatty acids derived largely from the metabolism of its blubber lipids [54]. By sampling during the first two months of breeding (January and February), we expected that the isotopic composition of gray whale milk was determined by patterns that were changing little with time, since they reflected those of prey consumed only during the feeding season. The same logistical constrains related to milk sampling are true for the measurement of fetus epidermis δ^15^N values (calf δ(0)). In addition, no information is available for the mother-to-fetus isotope fractionation in cetacean epidermis. Previous results suggest mother-to-fetus nitrogen isotope difference of ~1‰ for blood, plasma, and hair in other mammals such as elephant seals and humans [21,48], and of ~1.5‰ in fin whale’s muscle [29]. Interestingly, the two methods used here to estimate the highest mother-to-fetus nitrogen isotope fractionation gave results consistent with both literature values and resulted in little differences in the estimates of calf age (Fig 5). We consider “method 2” as the most conservative estimate to evaluate the physiological stage of each examined mother-calf pair because it does not assume that all whales have the same metabolic condition. Nevertheless, we suggest caution in interpreting isotopic clock results using the maximum δ^15^N_calf-mother_ if sampled calves are born within few days of sampling. In that case, SB δ^15^N values would differ little from the other epidermal layers, and predictions could result in a discrepancy called “the edge-effect bias” [55]. This could be the reason why younger birth dates were found for 2019 calves modelled using “method 2” compared to “method 1” (Fig 5, S1 File).

We did not observe calf δ^15^N values at steady state with maternal milk (calf δ(∞)) from direct sampling, because calf weaning occurrs in the polar feeding areas months after their stay in the breeding lagoon. For this reason, we estimated calf δ(∞) using information available in the literature for other species mother-calf pairs for which δ^15^N differences between mother and calf was known at a specific time after birth [47]. Jenkins et al [44] observed δ^15^N values of plasma and red blood cells were similar 12 to 14 days after parturition in those mother-calf pairs. Nothing similar has been reported in marine mammals, however, we assumed these data could be relevant for two reasons. First, due to the peculiar histology of its structural layers, whale epidermis combines turnover rates that vary from a few days to months [13,14,39], as do plasma and red blood cells [22,41]. Cells’ lifespan and isotopic turnover rates are unknown for the epidermis of gray whale, however, epidermal cell turnover rates are known for bottlenose dolphins [39] and beluga whales [40], and isotopic turnover is known for bottlenose dolphins [11] and blue whale [13] epidermis. These studies showed that while body size, body mass, feeding habits and habitat use of the analyzed animal differed, isotopic turnovers were comparable. Second, we observed a general decrease in calf δ^15^N values from SC to SB, while the nitrogen isotope composition of mothers’ epidermis was less variable. In all mother-calf pairs collected in 2018 and in 3 couples from January 2019, δ^15^N values were similar at a certain point (Fig 5B). The fact that equal δ^15^N values were found among different epidermal layers could be associated with the timing of calf birth, and the transition from gestation to lactation in the δ^15^N values of their epidermis. It is plausible that calf epidermis integrates quicker the nitrogen isotopic composition of maternal milk whereas mothers’ δ^15^N values record the physiological change from gestation to lactation [7]. Isotopic incorporation in body tissues is expected to be primarily influenced by protein turnover rate [56], which changes with temperature, body size and growth [57]. It is not known if the transition from mother’s womb to marine life has any effect on gray whale calf epidermis cells and isotopic incorporation rates. Both these parameters could be expected to have a faster rate in calves than in lactating females, because calves are much smaller than their mothers and are experiencing rapid growth [34]. Based on the similar results reported by others [11,13,40], epidermis δ^15^N values appear little affected by these variables, but the full complexity of protein turnover is still poorly understood and more field studies on animals of different age classes are needed.

### Implications and conclusions

Our model represents a potential method to estimate calf age based on mother-calf pairs epidermis δ^15^N values in capital breeders. We recognize our important assumptions based on previous studies that did not involve the gray whale. Nevertheless, the isotopic clock model here presented can be interpreted as preliminary, easily adjustable with new data. In this regard, future sampling campaigns should collect epidermis tissues from both live and stranded organisms. Every year, inside and outside the breeding lagoon of Baja California, and along the gray whale migratory corridors, many individuals wash ashore due to different reasons (e.g. sickness, starvation, boat accidents) [58]. A systematic monitoring and collection program of those carcasses could allow milk sampling from lactating mothers, or collection of epidermal tissues from the fetus and the dead pregnant females. Simultaneously, efforts should focus on marking pregnant females, to be able to obtain consecutive and time-related epidermal δ^15^N values from them and their calves, from birth to weaning.

The eastern gray whale is a good model animal and variation among its epidermal layer δ^15^N values during breeding have the potential to be useful for the study of other baleen whales during the same life-history phase. The western gray whale is listed as an endangered population by the IUCN. A precise estimate of gray whale calf birth dates among different years would allow the detection of possible alterations in the known patterns of the species’ reproduction and to immediately investigate the correlated causes. Additional studies are necessary to monitor changes with time in δ^15^N values of both mothers and calves. By collecting epidermis biopsies at the beginning and at the end of their stay in the study area, it may be possible to gain insights on the isotopic patterns of milk. Moreover, it may help to corroborate the higher expected turnover rate in calf epidermis, as well as the persistence of pregnancy effects on a mother’s δ^15^N value. Future investigation of feeding dynamics of pregnant and lactating baleen whale individuals emerges as critical. The success of their prolonged reproductive cycles varies according to the energy balance that exists between accumulation of nutrients and their use to supply specific physiological needs. In a time in which ethic statements strongly influence the management of conservation, the findings of this study could help to go beyond questionable and old-fashioned practices, such as scientific whaling. Non-lethal collection of epidermis biopsies and successive stable isotope analysis provide an effective tool for the study of reproduction, feeding, and population dynamics of migratory aquatic species.

## Supporting information

S1 File (XLSX)
